# An uncommon t(9;11)(p24;q22) with monoallelic loss of *ATM* and *KMT2A* genes in a child with myelodysplastic syndrome/acute myeloid leukemia who evolved from Fanconi anemia

**DOI:** 10.1186/s13039-018-0389-x

**Published:** 2018-07-11

**Authors:** Viviane Lamim Lovatel, Daiane Corrêa de Souza, Tatiana Fonseca Alvarenga, Roberto R. Capela de Matos, Claudia Diniz, Marcia Trindade Schramm, Juan Clinton Llerena Júnior, Maria Luiza Macedo Silva, Eliana Abdelhay, Teresa de Souza Fernandez

**Affiliations:** 1 Bone Marrow Transplatation Center (CEMO), National Cancer Institute (INCA), Rio de Janeiro, Brazil; 2 Pathology Department of National Cancer Institute (INCA) and Post-Graduation Program in Medical Sciences, Rio de Janeiro State University, Rio de Janeiro, Brazil; 3 Hematology Department, National Cancer Institute (INCA), Rio de Janeiro, Brazil; 4 Medical Genetic Departament, Fernandes Figueira National Institute, Oswaldo Cruz Foundation, Rio de Janeiro, RJ Brazil; 5 Post-Graduate Program in Oncology, National Cancer Institute José de Alencar Gomes da Silva (INCA), Rio de Janeiro, Brazil

**Keywords:** Myelodysplastic syndrome, Acute myeloid leukemia, Fanconi anemia, Classical cytogenetics, FISH, T(9;11), *ATM*, *KMT2A*

## Abstract

**Background:**

Myelodysplastic syndrome (MDS) is rare in the pediatric age group and it may be associated with inheritable bone marrow failure (BMF) such as Fanconi anemia (FA). FA is a rare multi-system genetic disorder, characterized by congenital malformations and progressive BMF. Patients with FA usually present chromosomal aberrations when evolving to MDS or acute myeloid leukemia (AML). Thus, the cytogenetic studies in the bone marrow (BM) of these patients have an important role in the therapeutic decision, mainly in the indication for hematopoietic stem cell transplantation (HSCT). The most frequent chromosomal alterations in the BM of FA patients are gains of the chromosomal regions 1q and 3q, and partial or complete loss of chromosome 7. However, the significance and the predictive value of such clonal alterations, with respect to malignant progress, are not fully understood and data from molecular cytogenetic studies are very limited.

**Case presentation:**

A five-year-old boy presented recurrent infections and persistent anemia. The BM biopsy revealed hypocellularity. G-banding was performed on BM cells and showed a normal karyotype. The physical examination showed to be characteristic of FA, being the diagnosis confirmed by DEB test. Five years later, even with supportive treatment, the patient presented severe hypocellularity and BM evolution revealing megakaryocyte dysplasia, intense dyserythropoiesis, and 11% myeloblasts. G-banded analysis showed an abnormal karyotype involving a der(9)t(9;11)(p24;q?22). The FISH analysis showed the monoallelic loss of *ATM* and *KMT2A* genes. At this moment the diagnosis was MDS, refractory anemia with excess of blasts (RAEB). Allogeneic HSCT was indicated early in the diagnosis, but no donor was found. Decitabine treatment was initiated and well tolerated, although progression to AML occurred 3 months later. Chemotherapy induction was initiated, but there was no response. The patient died due to disease progression and infection complications.

**Conclusions:**

Molecular cytogenetic analysis showed a yet unreported der(9)t(9;11)(p24;q?22),der(11)t(9;11)(p24;q?22) during the evolution from FA to MDS/AML. The FISH technique was important allowing the identification at the molecular level of the monoallelic deletion involving the *KMT2A* and *ATM* genes. Our results suggest that this chromosomal alteration conferred a poor prognosis, being associated with a rapid leukemic transformation and a poor treatment response.

## Background

Myelodysplastic syndrome (MDS) comprises a heterogeneous group of clonal neoplastic blood diseases characterized by ineffective hematopoiesis, peripheral cytopenias, bone marrow dysplasias and an increased risk of acute myeloid leukemia (AML) [1]. Pediatric MDS is an uncommon disorder accounting for 4–9% of hematologic malignancies [2, 3] and it may be associated with inherited bone marrow failure (BMF) disorders such as Fanconi Anemia (FA) [4].

First described in 1920 by the pediatrician Guido Fanconi [5], nowadays, it is known that FA is a cancer-prone chromosomal instability disorder with diverse clinical symptoms. The congenital anomalies may include skeletal defects, classically abnormal thumb or radius, short stature, café-au-lait spots and endocrinopathies. FA is a rare autosomal and X-linked genetic disease, with a wide variety of symptoms, characterized by congenital abnormalities, progressive BMF and increased cancer risk, which can be difficult to diagnose [5–7].

At the molecular level, 21 FA-related genes that constitute FA-BRCA pathway were identified [6]. Proteins encoded by FA-related genes play important roles in various cellular functions, including DNA repair, detoxification of reactive oxygen species and aldehydes, energy metabolism and both pro-inflammatory and myelosuppressive cytokine homeostasis [8].

Since the first clinical report of FA, important laboratory and clinical advances were incorporated in the diagnosis and treatment, such as the DEB test and the application of HSCT, with a low dosage chemotherapy regimen for children with FA [9–11].

Supportive care using hematopoietic growth factors such as EPO, G-CSF or androgens such as oxymetholone to boost blood cell production, provides transient benefit in some patients. Although, the allogeneic HSCT remains the main treatment approach to advanced marrow failure in FA patients [11, 12]. In this context, some preclinical studies using gene therapy in an attempt to improve the life quality and survival of patients with FA have been performed [12].

Cytogenetic studies in the bone marrow have an important role in the therapeutic decision, mainly in the indication for HSCT. A better understanding of the clinical relevance and biological implications of clonal chromosomal alterations in FA patients was achieved over the last decade by the incorporation of molecular cytogenetic technologies in addition to classical karyotyping [11, 13]. This has led to the identification of some specific chromosomal alterations in FA patients, their prognostic value and association with the risk of evolution to MDS and/or AML. The most frequent chromosomal alterations in bone marrow of patients with FA are gains of the chromosomal regions 1q and 3q, and partial or complete loss of chromosome 7. Besides, monosomy 7 and del(7q) have been associated with poor prognosis and progression to leukemia. A few reports also suggested that gains of 3q are associated with progression to MDS/AML and overall presenting poor prognosis [13, 14].

Alterations involving del(11q) are uncommon in FA and MDS, so the prognostic value of this alteration remains uncertain [13, 15]. In this region, there are genes such as Ataxia Telangiectasia Mutated gene [*ATM* (11q22)] and lysine methyltransferase 2A [*KMT2A* (11q23)] that have been described as having an important role in the pathogenesis of MDS. The *ATM* gene acts on the regulation of the cell cycle after a DNA damage is recognized [15, 16]. On the other hand, the *KMT2A* gene encodes a protein that is involved in chromatin remodeling and positively regulates multiple homeobox transcription factors, also it is highly associated with the development of AML [17].

Given the high incidence of hematological complications of FA patients, BM surveillance for morphological and cytogenetic changes provides an important contribution to the clinical decision [11]. However, there are only a few studies in patients with FA showing the bone marrow chromosomal alterations analyzed by classical and molecular cytogenetics associated with evolution to MDS and AML [13, 14, 17–19]. Here, we describe an uncommon yet unreported t(9;11)(p24;q22) with monoallelic loss of *ATM* and *KMT2A* genes, defined by classical cytogenetic and FISH analysis, in a child with MDS/AML who evolved from FA associated with poor clinical outcome.

## Case presentation

**Fig. 1:**
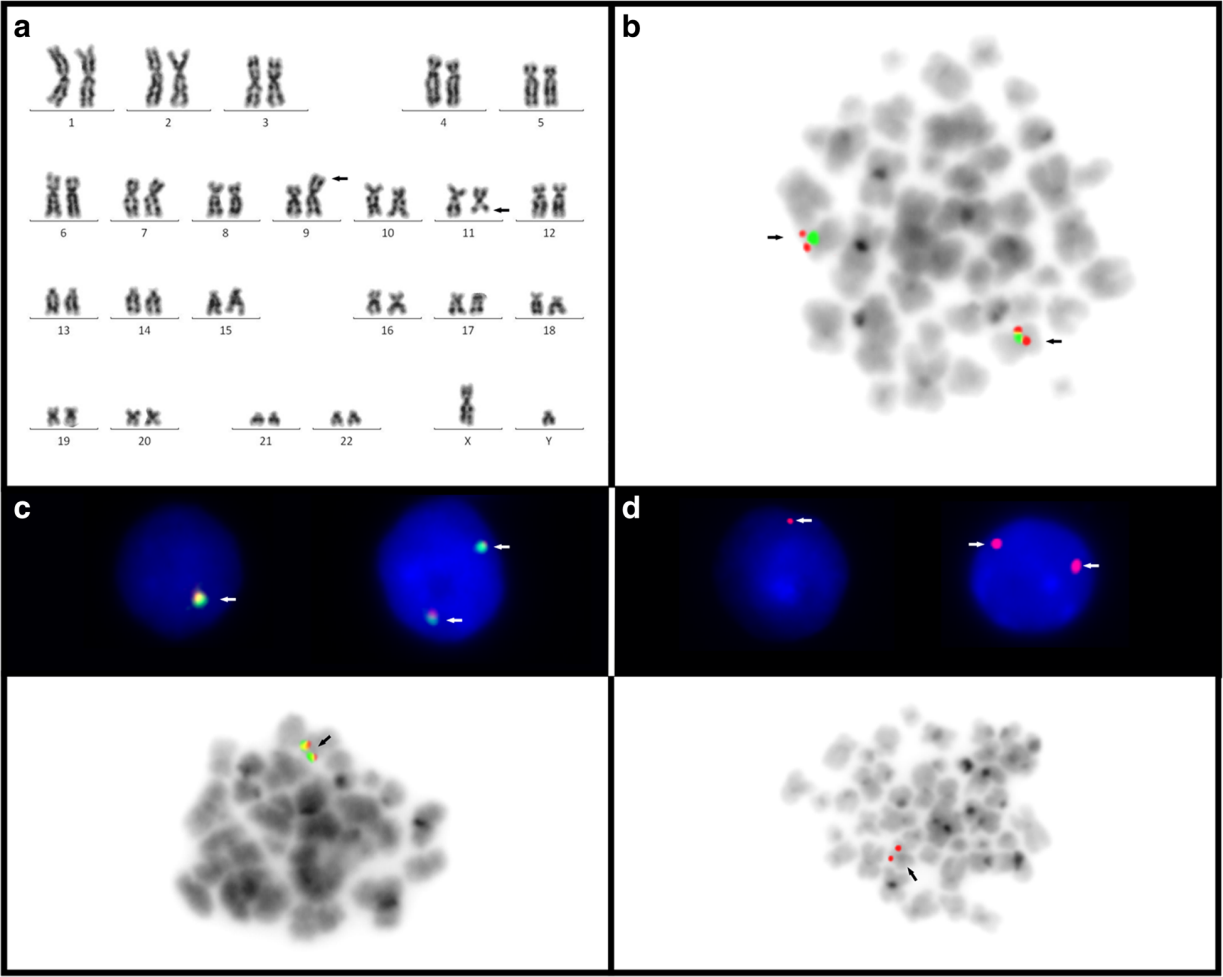
**a** G-banded showing the der(9)t(9,11)(p24;q?22). The black arrows point the gain of chromosome material on 9p and the loss on 11q; **b** FISH analysis using LSI *p16* probe [LSI p16 (9p21), red signal/CEP 9 (9p11-q11), green signal, Dual Color Probe, Vysis] and inverted DAPI, showing that *CDKN2A* gene was normal in a metaphase. The black arrows shows both CDKN2A normal signals; **c** FISH analysis using LSI *KMT2A* Dual Color break apart rearrangement probe Vysis, showing the monoallellic loss of the *KMT2A* gene with the loss of one signal in interphase nuclei (white arrows) and in metaphase using inverted DAPI (black arrow). **d** FISH using LSI *ATM* (11q22) spectrum orange probe, Vysis showing the monoallellic loss of *ATM* with the loss of one signal in interphase nuclei (white arrows) and in metaphase using inverted DAPI (black arrow)

A five-year-old male patient with recurrent infections and persistent anemia was admitted at the National Cancer Institute, Rio de Janeiro, Brazil. Physical examination showed a small stature (<P2); hyperpigmentation around the eyes; enophthalmia; multiple cafe-au-lait spots; hypoplasia of the thenar eminence accompanies left thumb hypoplasia. Laboratory findings: Hb 9.1 g/dl (age-adjusted 13.5–18.0 g/dl), platelet count 40 × 10^9^/l (150-400 × 10^9^/l) and white blood cell count 7.6 × 10^9^/l (age-adjusted range 4-10 × 10^9^/l). BM findings: hypocellularity and normal karyotype by G-banding, according to the International System form Human Cytogenomic Nomenclature (ISCN 2016) [20]. Clinical genetic exams were done at Medical Genetic Department, Fernandes Figueira National Institute, Oswaldo Cruz Foundation, Rio de Janeiro, Brazil. Chromosome breakage test cytogenetic analysis was performed in peripheral lymphocytes during 72 h of cultures exposed to DEB (0.1 μg/ml), according to Auerbach [9]. This analysis demonstrated spontaneous chromosome breakage - 0.16 breaks per cell (reference 00.00–0.08) and DEB-induced chromosome breakage - 2,32 breaks per cell (reference 0.00–0.08), confirming the FA diagnosis. He was hospitalized due to the persistent anemia and progressive neutropenia. Oxymetholone (50 mg/day) was the initial treatment. A partial hematological response was achieved, even with oxymetholone dose reductions due to liver toxicity. However, the hematological parameters worsened and erythropoietin (EPO) and granulocyte-colony stimulating factor (G-CSF) were associated, as well as danazol 200 mg/day was introduced. He also received multiple blood transfusions during the treatment but no satisfactory response was achieved. At this time, BM evaluation revealed dysplastic megakaryocytes, intense dyserythropoiesis and 11% of myeloblasts. Immunophenotypic analysis of BM cells also revealed 11% of myeloblasts expressing CD34/CD13/CD11b (54.25%), HLA-DR/CD33/CD7 (31,54%), dysplastic erythropoiesis (CD36/CD71/CD235a), dysgranulopoiesis (CD13/CD16/CD11b/Cd33/CD64/CD15,CD45) and monocytic lineage expressing CD14/CD64/CD36/HLA-DR,CD45. Cytogenetic analysis with G-band technique in bone marrow cells showed an abnormal karyotype: 46,XY,der(9)t(9;11)(p24;q?22)[9]/46,XY[12] (Fig. 1a). The diagnosis was MDS, refractory anemia with excess of blasts (RAEB). Fluorescence in situ hybridization (FISH) was performed to analyze some genes that may be altered during chromosomal rearrangement. So, we investigated the *CDKN2A* gene (located in 9p) and the *ATM* and *KMT2A* genes (located in 11q22 and 11q23, respectively), due to the important role they play during leukemogenesis. The FISH analysis for the *CDKN2A* gene showed two normal signals (Fig. 1b). It was observed a monoallelic loss of *KMT2A* and *ATM* genes (Fig. 1c and d, respectively). The final karyotype with G-banded and FISH analysis, according to the ISCN 2016 [20], was: 46,XY,der(9)t(9;11)(p24;q?22),der(11)t(9;11)(p24;q?22)[9].ish del(11)(q22.3q23)(ATM-)(KMT2A-)[7]. Treatment with decitabine was initiated and well tolerated, although progression to AML occurred 3 months later. He had no remission with systemic chemotherapy. The patient died 8 months after the diagnosis of RAEB-t, due to disease progression and infectious complications. Allogeneic HSCT was indicated early in the diagnosis, but non-consanguineous parents were available and no donor was found.

## Discussion and conclusions

Bone marrow failure occurs with a median age of 8 years and remains the primary cause of morbidity and mortality in FA [12]. The FA cells have a reduced fidelity in processing DNA double-strand breaks. This specific intrinsic susceptibility might, together with extrinsic factors, influence the course of the disease, resulting in the outgrowth of clones with chromosomal aberrations in the bone marrow cells [6]. Nevertheless, there is a cytogenetic clonal fluctuation in patients with FA, since clones often can disappear, reappear, evolve or be replaced by entirely new clones [10]. Therefore, the significance and the predictive value of such clonal alterations with respect to malignant progress are not fully understood [6].

Rochowski et al., (2012) hypothesized that unique bone marrow cytogenetic clones may distinguish patients with FA and AML from patients with de novo AML. Some chromosomal abnormalities as the gain of 1q, 3q, 13q and partial loss of 7q, 20q, 11q or complete loss of chromosome 7 are more frequent in patients with FA who showed progression to AML. While others like trisomy 8, t(8;21), t(9;11), t(6;9) and inv.(16) have been described exclusively in patients with de novo AML [21]. In this study, the t(9;11) was described in MDS/AML secondary from FA. Nevertheless, it is important to note that the breakpoints involved in this chromosomal translocation were not yet reported [22, 23]. In literature, the recurrent breakpoints involved in the t(9;11) in de novo AML patients were: t(9;11)(q34;q23) with *FNBP1/KMT2A* gene rearrangement [24]; t(9;11)(q34;p15) with *NUP98/PRRX2* [25]; t(9;11)(q34;q23) with *AF9q34/MLL* [26] t(9;11)(p22;p15) with *NUP98/PSIP1* [27]; and t(9;11)(p21;q23) with *MLLT3*/*KMT2A* [28]; t(9;11)(q34;q23) with *DAB2IP/KMT2A* [29].

In our study, the patient with FA showed the acquisition of a der(9)t(9;11)(p24;q?22), der(11)t(9;11)(p24;q?22) associated with evolution from MDS to AML. In FISH analysis, it was detected the monoallelic loss of *ATM* gene. The *ATM* gene is located at 11q22 region. This gene encodes a serine/threonine protein kinase, a critical enzyme in the regulation of the stress response to DNA damage, especially double-strand DNA breaks and it is also involved in cell cycle control. The *ATM* gene appears to act as a “caretaker” of the genome [16]. The loss of *ATM* has been described as having contributed to the increased need for transfusion in patients with MDS associated with del(11q) [15]. Currently, it has been demonstrated the joint and reciprocal action of *ATM* and FA proteins in the DNA repair pathway during replication [30, 31].

In our study, the FISH analysis also showed the monoallelic loss of the *KMT2A* gene. The *KMT2A* locus is involved in more than 60 different chromosomal translocations in pediatric acute leukemias [17]. In AML, the t(9;11) usually results from *KMT2A* gene translocation, being the (p22;q23) the breakpoint frequently described [11]. Interestingly, it was detected the monoallellic deletion of *KMT2A* gene in our study. Wang et al. showed that deletions of chromosome 11q lack cryptic *KMT2A* rearrangements in patients with MDS and suggested that loss of tumor suppressor genes located in 11q through deletions and secondary allelic loss of heterozygosity is likely more important in pathogenesis and disease progression [15]. The detection of all possible types of *KMT2A* cyto-molecular abnormalities is of key importance for the identification of biological subgroups, which may differ in clinical outcome [30].

In the literature, data on FISH studies in FA are quite limited [14]. In our study, the analysis by the FISH technique was essential allowing the identification at the molecular level of the monoallelic deletion involving the *KMT2A* and *ATM* genes. With the FISH results it was possible to note that this chromosomal abnormality was not a simple reciprocal translocation involving the regions of chromosomes 9p24 and 11q22, but an unbalanced chromosomal alteration due to the monoallelic loss of *ATM* and *KMT2A* genes. FISH continues to be considered an important technique for molecular investigation of small or hidden chromosomal abnormalities [32].

Taken together, the monoallelic deletions of the genes *KMT2A* and *ATM*, probably, contributed to the defects in the genomic instability of the chromatin remodeling, conferring a poor prognosis, being associated to a rapid leukemic transformation and a poor response to treatment. Furthermore, with our results we contribute to the literature showing an yet unreported der(9)t(9;11)(p24;q?22),der(11)t(9;11)(p24;q?22) with monoallelic loss of *ATM* and *KMT2A* genes.

## Abbreviations

AA: Aplastic anemia
AML: Acute myeloid leukemia
*ATM*: Ataxia Telangiectasia Mutated gene
BM: Bone marrow
BMF: Bone marrow failure
*CDKN2*: Cyclin-dependent kinase inhibitor 2A
*DAB2IP* DAB2: Interacting protein
DEB: Diepoxybutane
EPO: Erythropoietin
FA: Fanconi anemia
FA-BCRA DNA: Repair associated
FANC: Fanconi anemia complementation
FISH: Fluorescence in situ hybridization
*FNBP1*: Formin binding protein 1
G-CSF: Granulocyte-colony stimulating factor
HSCT: Hematopoietic stem cell transplantation
ICLs DNA: Interstrand cross-links
*JAK*: Janus kinase 2
*KMT2A*: Lysine methyltransferase 2A
MDS: Myelodysplastic sydrome
*MLLT3*: Super elongation complex subunit
MMC: Mitomycin C
*NUP98*: Nucleoporin 98
*PRRX2*: Paired related homeobox 2
*PSIP1*: PC4 and SFRS1 interacting protein 1
RAEB: Refractory anemia with excess of blast
